# Citizenship formation and resilience among Ukrainian female migrants: case studies from Norway

**DOI:** 10.3389/fpsyg.2025.1720857

**Published:** 2026-01-07

**Authors:** Miroslava Tokovska, Signe Alexandra Domogalla, Ashley Rebecca Bell-Mizori, Vanessa Nolasco Ferreira

**Affiliations:** 1 Department of Health and Exercise, School of Health Sciences, Kristiania University of Applied Sciences, Oslo, Norway; 2 Department of Performing Arts, School of Arts, Design and Media, Kristiania University of Applied Sciences, Oslo, Norway; 3 Department of Psychology, Pedagogy and Law, School of Health Sciences, Kristiania University of Applied Sciences, Oslo, Norway; 4 Department of Psychology, Pedagogy and Law, School of Health Sciences, Kristiania University of Applied Sciences, Bergen, Norway

**Keywords:** narrative exposure therapy, migration, PTSD, citizenship formation, resilience

## Abstract

**Introduction:**

The escalation of the armed Russian/Ukraine conflict in 2022 precipitated a significant humanitarian crisis. The ensuing forced migration, trauma, and family separation presented complex challenges, particularly for women. This study aims to understand complex social phenomena through a detailed examination of how Ukrainian female refugees in Norway navigate citizenship formation and develop resilience strategies while in transit.

**Methods:**

Utilizing a collective case study approach, the research that was anchored in Narrative Exposure Therapy (NET) collected, as part of the treatment approach, narratives from six Ukrainian female refugees in Norway. Narrative and thematic analysis were employed on the data, which was interpreted through the theoretical frameworks of citizenship and resilience.

**Results:**

The study revealed citizenship as a dynamic, multidimensional process characterized by strategic institutional engagement, identity reconstruction, and adaptive resilience. Participants demonstrated a remarkable capacity to transform refugee status from a passive categorization to an active process of belonging. Key mechanisms included leveraging professional identities, maternal experiences, and emotional adaptation strategies.

**Discussion:**

This research provides multidimensional insights into forced migration experiences, highlighting the complex interplay between individual agency and institutional support. It challenges traditional understandings of citizenship, emphasizing resilience as a continuous, context-dependent process of negotiation and adaptation.

## 1 Introduction

The armed conflict between Russia and Ukraine, escalated by Russian tanks crossing the border on February 24, 2022, triggered a humanitarian crisis. This has as consequence implications for health systems across Europe, which were still recovering from the disruptions brought about by the COVID-19 pandemic (Leon et al., 2022; World Health Organization, 2025). Since the beginning of the conflict ongoing research has shown that Ukrainian women face distinct psychological risks related to forced displacement, caregiving responsibility, and war-related trauma. Studies conducted among refugee women and internally displaced mothers consistently report high levels of stress, anxiety, traumatic exposure, and emotional strain while navigating unstable and often unfamiliar environments demonstrating that Ukrainian women's mental health outcomes are influenced not only by direct exposure to violence but also by prolonged uncertainty, separation from family members, and the burden of supporting children during displacement (Adedeji et al., 2025; Baran et al., 2024; Preiss et al., 2024).

Gender-based violence and caregiving duties in unstable environments increased the vulnerability of women still further (Kirkbride et al., 2024). Anjum et al. (2025) shows that mothers under collective protection policies experience persistent worry for relatives remaining in Ukraine, disrupted attachment processes, and difficulty re-establishing a sense of safety in host countries. Similar findings emerge among pregnant and postpartum Ukrainian women, who report heightened fear, traumatic stress, and concerns about raising children while living in uncertainty (Krupelnytska and Morozova-Larina, 2025; Krupelnytska et al., 2025). Perinatal and early-parenting studies emphasize that the war has intensified psychological vulnerability across motherhood stages, affecting women's identity reconstruction and caregiving capacity amid displacement (Rodríguez-Muñoz et al., 2023; Chrzan-Detkoś et al., 2022; Chrzan-Detkoś and Murawska, 2023). The scale of the forced migration created challenges for the social and healthcare infrastructures of the countries that received the refugees.

Norway has taken substantial action in response. The (Norwegian Directorate of Immigration (UDI), 2025b) granted collective protection to 90,766 Ukrainian refugees out of 92,441 applicants (as of June 6th, 2025). The refugee influx occurred in two distinct waves: 35,920 arrivals in 2022 and 36,461 in 2023, with eligibility contingent upon documented residence in Ukraine when the war broke out February 2022 (Norwegian Directorate of Immigration (UDI), 2025a). The initial refugee population as of June 2022 exhibited a significant gender imbalance, with women comprising 79% of arrivals—a distribution that directly reflected Ukraine's wartime conscription policies restricting male departure (Hernes et al., 2025). In the described scenario it is important to highlight that Ukrainian women in Norway shows that uncertainty related to temporary protection, caregiving alone in a new country, and feelings of suspended life plans contribute to chronic emotional strain and difficulties with long-term integration (Anjum et al., 2025). As Ukrainians have grown to become the largest refugee group in Norway, the country has modified its policies to manage public service demands in housing, healthcare, and education sectors, including more selective protection measures for individuals from designated safe regions in Ukraine (Government of Norway, 2024).

These unprecedented migration flows have necessitated a fundamental reconsideration of how societies adapt to and integrate large numbers of forced migrants. The last decade of research has highlighted how traditional conceptualizations of citizenship are challenged by transnational forced migration patterns, with the identity of forced migrants heavily dependent on host society recognition (Bauloz et al., 2019; Espinoza and Muñoz, 2025). This understanding has evolved beyond viewing citizenship as merely a legal framework; it is recognized as a dynamic process that significantly influences refugees' resilience trajectories (Favell, 2022). Building on this evolution, Sobhy (2024) proposes a comprehensive framework for understanding lived citizenship through four key fields—protection, provision, participation, and belonging—which are critical for examining how forced migrants navigate their relationship with the state. The interplay between these fields' shapes both individual adaptation processes and broader social integration outcomes. Recent studies demonstrate that, while secure legal status correlates with improved mental health outcomes and social integration (Ermansons et al., 2023), forced migrants actively construct resilience through informal citizenship practices (Kraler et al., 2022; Qamar, 2023), even as they navigate challenging immigration environments (Berding-Barwick and Mcareavey, 2024).

Despite the scale of the Ukrainian displacement, psychological research explicitly examining women's lived experiences during and after the 2022 invasion remains ongoing and new data arise every day. However, the existing studies clearly show that Ukrainian women negotiate displacement through encrusted processes of trauma recovery, caregiving demands, and identity reconstruction under conditions of uncertainty (Baran et al., 2024; Preiss et al., 2024; Krupelnytska et al., 2025). These insights highlight the importance of grounding analyses of citizenship formation and resilience in the gendered realities of forced migration to contribute with the understanding about how Ukrainian women in Norway navigate these interwoven psychological and social processes.

Given the complexity of these dynamics and their implications for both individuals and host societies, this study aims to understand complex social phenomena through a detailed examination of how Ukrainian female refugees in Norway navigate citizenship formation and develop resilience strategies during forced migration. The Norwegian context provides a unique opportunity to examine how forced migrants navigate both formal and informal pathways to belonging and integration. Building on this evolving understanding of citizenship and resilience in forced migration contexts, this research addresses the question:

How do Ukrainian female refugees in Norway navigate processes of citizenship formation and develop resilience strategies in the context of forced migration following the 2022 Russian-Ukrainian conflict?

### 1.1 Theoretical framework: conceptualizing citizenship and resilience in forced migration

The concept of citizenship in forced migration contexts requires a nuanced theoretical understanding that goes beyond traditional legal-political frameworks. Contemporary citizenship theory has evolved from Marshall and Bottomore (1950) classical conception of citizenship as comprising civil, political, and social rights, to a more complex understanding that encompasses the cultural, emotional, and transnational dimensions of belonging and participation. In the context of forced migration, citizenship can be understood through what Isin (2009) and Isin and Nielsen (2008) term “acts of citizenship” —whereby individuals enact themselves as citizens through various practices and claims-making, irrespective of their formal status. Using this framework, this study explores how Ukrainian female refugees adapt and exercise agency in Norway's new social and political environments. As Nyers (2015) argues, refugees are not passive recipients of rights but active agents in constructing new forms of political subjectivity. The concept of emotional citizenry, as described by Askins (2016), offers a crucial theoretical lens by recognizing that citizenship extends beyond legal status to encompass deep emotional attachments and psychological adaptation processes. This framework is particularly relevant for understanding how trauma and displacement influence the formation of citizenship among forced migrants. According to Fransen-Jaïbi et al. (2021), the concept of participatory citizenship emphasizes how citizenship is enacted through various social and political participation forms, focusing on practices that support citizens' social and cultural integration in the local community. In this study, the concepts of emotional citizenry and participatory citizenship help to explain how Ukrainian women have constructed citizenship through formal and informal engagement with their host society.

The concept of resilience in forced migration contexts has evolved significantly from the early psychological definitions that focused on individual traits; the more recent complex socio-ecological interpretation recognizes resilience as a dynamic process shaped by multiple factors and systems (Berding-Barwick and Mcareavey, 2024; Qamar, 2023). The resilience theory in forced migration studies builds upon Ungar (2011) socio-ecological model, which conceptualizes resilience as both an individual's capacity to navigate toward resources that sustain wellbeing and the capacity of the individual's environment to provide these resources in culturally meaningful ways. The present perspective effectively captures how Ukrainian refugees navigate new social contexts while maintaining cultural connections.

Sisto et al. (2019) define psychological resilience as the ability to maintain one's orientation, despite difficulties, toward existential purposes; it is characterized by perseverance, self-awareness, and internal coherence in service of personal growth. On the other hand, Lenette et al. (2013) emphasize that refugee resilience must be understood as a continuous process of negotiation rather than a fixed outcome. Their work highlights how refugees demonstrate everyday resilience through routine practices and their daily negotiations of new environments. The outlined approach recognizes that resilience in the context of forced migration often involves combining pre-existing capabilities with new adaptive strategies developed in response to displacement. This lens proves particularly relevant for understanding how Ukrainian female refugees utilize both pre-war professional identities and newly developed skills in their adaptation processes.

The interplay of citizenship and resilience in forced migration has unveiled intricate dynamics between legal status, identity formation, and adaptive capacities. These theoretical perspectives collectively provide a comprehensive framework for understanding citizenship as a dynamic and multi-dimensional process. By examining citizenship through these diverse theoretical perspectives, we can achieve a more nuanced analysis of how Ukrainian female refugees adapt to their new lives in Norway, construct new forms of belonging, and sustain transnational ties. This holistic approach elucidates how forced migrants navigate the intricate processes of belonging and participation in their new environments while maintaining connections to their homeland. In addition, this theoretical framework posits that citizenship should be perceived not as a fixed status, but as an evolving process of becoming, manifested through various practices, claims, and forms of participation.

## 2 Materials and methods

### 2.1 Research design

The research design follows a collective case study approach (Tracy, 2024), examining multiple individual cases to identify broader patterns while preserving attention to unique experiences, analyzing data across micro- (interactional), meso- (organizational), and macro- (societal/cultural) levels to generate insights that inform policy, community action, or professional development. This design allows for both within-case analysis and cross-case comparison, enabling the identification of both unique and common elements across participants' experiences (Crowe et al., 2011). The case study methodology was selected for its ability to provide a rich, contextual understanding of complex social phenomena (Priya, 2021) and its suitability for examining lived experiences in forced migration contexts. The pattern analyzed here is connected to citizenship formation among Ukrainian women treated with Narrative Exposure Therapy (NET) in the ongoing feasibility study “FactArt: The Society Sees Life Narratives.” Therefore, only narratives in which this pattern emerged were included in the present article, following the premises of qualitative studies (Palinkas et al., 2015).

### 2.2 Study context

“FactArt: The Society Sees Life Narratives” is a project, of which this study is a part, initiated in 2023 at Kristiania University of Applied Sciences in response to the growing need for Ukrainian citizen integration into Norwegian society. The research was conducted through interdisciplinary collaboration across psychology, public health, social sciences, and performing arts with three main objectives: (1) to generate new knowledge about PTSD symptom treatment using NET; (2) to develop a methodology for creating psychoeducational artistic interventions using collective composite narratives derived from individual treatment sessions with survivors; and (3) to collect physiological biomarkers (heart rate variability) to inform future studies and improve project interventions.

NET is an evidence-based intervention that can treat symptoms connected to PTSD due to exposure to organized violence. NET is a short-term therapeutic approach designed to help individuals process and integrate traumatic memories by constructing a coherent narrative of their life experiences (Schauer et al., 2025). Elbert et al. (2022) demonstrate the efficacy of NET in reducing PTSD symptoms among refugees and asylum seekers, highlighting its potential applicability for displaced Ukrainian women. The therapy involves detailed recounting of traumatic events in chronological order, which helps to contextualize and diminish the emotional impact of these memories based on the premise of double representation of memory. The nature of the therapy, described by Costanza et al. (2022), also allows the possibility of building social context around traumatization and resilience. Through individual therapy, which can mitigate trauma in refugees, the narrative can be used to design a psychoeducational intervention based on storytelling. This is then examined through qualitative methods and artistic methods from performing- and media arts, with the aim of discussing trauma, violence, and conflicts on a societal level.

In the context of this study, NET also served as a source of qualitative data. As documented in Kaltenbach et al. (2020), NET requires clients to narrate their life story chronologically while the therapist actively records the narrative session by session, capturing sensory, cognitive, emotional, and autobiographical elements of each event. Therapists then produce a written version of the narrative and read it back to the client for confirmation and continuation in the following session. Through this process, fragmented traumatic memories become organized into a coherent autobiographical document that reflects the individual's lived experiences and meaning-making processes. Because NET systematically elicits detailed, structured, and contextually grounded life narratives, these written testimonies can be used as robust qualitative material for narrative and thematic analysis, provided ethical safeguards and participant consent are ensured.

The narratives collected throughout this project were analyzed through citizenship and resilience frameworks to understand complex social phenomena, particularly focusing on the individual experiences of Ukrainian female refugees. The detailed examination provides insights into how displaced individuals navigate their new social environment while managing trauma responses.

### 2.3 Sampling and participant selection

Participants were recruited through a public announcement on the Kristiania University of Applied Sciences website, which included a link to an online contact form. In addition, a Ukrainian research assistant facilitated recruitment by connecting the team with Ukrainian refugee communities in and around Oslo, Norway, where the online contact form was also shared. Interested individuals completed the online contact form (*N* = 23) and were subsequently contacted by the research team. Those who met the inclusion criteria and expressed continued interest were invited to receive a mental health “pre-” screening (*N* = 15). The inclusion criteria specified that participants must be Ukrainians aged 18–55 who arrived in Norway as refugees after February 2022 and were able to speak one or more of the following languages: Norwegian, English, Ukrainian or Russian. Following the mental health screening, those classified as having PTSD were offered between 4–8 sessions of NET treatment (*n* = 12, all female). Participants with other severe mental health issues, such as psychotic spectrum or mood disorders, or participants already undergoing trauma treatment, were excluded or referred to the public health system.

The sampling strategy aligns with what Palinkas et al. (2015) term “maximum variation sampling,” allowing for the examination of both common patterns and unique variations in refugee experiences. Selection criteria included arrival in Norway post-February 2022, varied family situations (single, married, with and without children), and diverse pre-war locations within Ukraine, ensuring rich data collection across different displacement contexts and personal circumstances. This carefully constructed sample provided the foundation for an in-depth analysis of citizenship formation and resilience processes among Ukrainian female refugees.

For the present qualitative analysis, six of the 12 women who completed NET were selected because their life narratives contained substantial material directly related to the study's core analytical focus: processes of citizenship formation. These six participants described detailed experiences across the fields of protection, provision, participation, and belonging, allowing for systematic interpretation through the citizenship and resilience frameworks used in this study.

The remaining participants completed NET but did not provide narrative material that engaged directly with citizenship-related themes or lacked sufficient depth on issues such as institutional navigation, identity reconstruction, or social participation. For this reason, their narratives were not included in the qualitative analysis. This selection approach aligns with purposive, information-rich sampling strategies that prioritize conceptual relevance over numerical representation in qualitative case-based research (Palinkas et al., 2015; Levitt et al., 2018).

### 2.4 Data collection

Primary data was collected through NET sessions conducted in women who met the inclusion criteria. The implementation of NET in the study consistently involved two professionals: a therapist and an observer in the room with each participant. In addition, a government-approved interpreter was also present during the therapy sessions in cases where translation from English to Ukrainian or Russian was necessary, ensuring both therapeutic integrity and participant support. The therapy followed the structured approach of constructing a chronological narrative of the participants' life through the creation of a “lifeline” with symbols representing significant events, followed by detailed elaboration on these events across sessions, and culminating in a written testimony of the individual's complete life story.

The narratives were collected using the therapists' transcripts of the exposure sessions focusing on the participants' perspective, as predicted in the therapeutic method. Word-Dictaphone was used during these sessions to capture the narratives, enhancing the validity of the data collection, after it the transcripts were controlled ensuring the quality of the patient's auto-biographical testimonies. In connection with exposure techniques, the narratives were read to the participants at the beginning of the following session, allowing them to correct, change, or approve what was written. The final re-reading of the complete narrative took part in the concluding session, with participants having the opportunity to make their last adjustments. All narratives were written down in English.

### 2.5 Data analysis

The analysis followed a systematic approach combining elements of narrative analysis (Allen, 2017) and thematic analysis (Naeem et al., 2023). The process involved: (1) Initial coding, (2) Development of case narratives for each participant, (3) Thematic analysis across cases, and (4) Theoretical interpretation through citizenship and resilience frameworks. To ensure methodological rigor and research quality, this study implemented multiple validation strategies aligned with Ahmed (2024)'s criteria for trustworthiness in qualitative research. Validity was established through member checking, where in the re-reading therapeutic process, participants reviewed and verified their case narratives, while reliability was strengthened through systematic peer debriefing sessions with colleagues specializing in forced migration research. Objectivity and transparency were maintained through a detailed audit trail documenting all analytical decisions and methodological choices. The credibility of the study was further enhanced through data triangulation incorporating multiple sources, including discussion between researchers with various backgrounds and relevant documents. This comprehensive approach to research quality, combining rigorous validation procedures with transparent documentation, aligns with contemporary standards for qualitative research excellence while acknowledging the complex nature of forced migration studies. Quotes presented in Section 3. Results are slightly edited to improve readability, while remaining as close to the original statements as possible.

### 2.6 Ethical considerations

The project “FactArt: The Society Sees Life Narratives” complies with ethical guidelines for research that involves human participants, including obtaining informed consent and implementing measures to safeguard confidentiality and General Data Protection Regulation (GDPR). The project was approved by both the Regional Committee for Medical and Health Research Ethics (REK, reference no. 588073) and the Norwegian Agency for Shared Services in Education and Research (SIKT, ref. no. 662949), with the final approvement received November 18th, 2024, after adjustments in the timeline were made and approved by these committees. All the data were securely saved using Services for Sensitive Data (TSD), a platform for collecting, storing, analyzing, and sharing sensitive data in compliance with Norwegian privacy regulations. This secure data management system ensured participant confidentiality throughout the research process.

## 3 Results

The following case narratives present the experiences of six Ukrainian female refugees who fled to Norway following the February 2022 Russian-Ukrainian conflict. Their stories highlight themes of trauma, resilience, adaptation, and the complex journey of establishing new lives while processing displacement and loss. These narratives demonstrate how individual strength, support systems, pre-war trauma, family dynamics, and emotional processing collectively influence adaptation and citizenship development in refugee resettlement. Table 1 with participant's demographics and therapeutic context is presented as follow.

**Table 1:** Participant's demographics and therapeutic context.

**Participant**	**Age range**	**Region of origin**	**Arrival in Norway**	**Family status**	**Use of an interpreter**	**Completed NET sessions**
A	45–50	Eastern Ukraine	2022	Divorced, 2 children	Yes	8
B	30–35	Northwestern Ukraine	2022	Married, 1 child	Yes	7
C	35–40	North-Central Ukraine	2022	Single, no children	No	6
D	50–55	Central Ukraine	2022	Married, 2 children	Yes	8
E	35–40	Eastern Ukraine	2023	Divorced, 1 child	Yes	6
F	40–45	Northwestern Ukraine	2023	Divorced, 2 children	Yes	7

### 3.1 Participant A: from marriage to war—a journey of self-discovery

Participant A begins her narrative with her early marriage at the age of 18, after escaping from an abusive stepfather. Her initial marriage was marked by controlling behavior and domestic violence, reflecting patterns of trauma that would later influence her response to displacement. Her journey to Norway exemplifies both the chaos of war and the emergence of survival strategies. She describes taking practical steps, such as collecting cash daily from her store, in preparation for an uncertain future. The narrative captures the intense emotional and physical challenges of evacuation:

“*We were scared because during the whole period we were on the road there was a lot of shooting towards the road... That was very scary.”* (PA)

Her interaction with Norwegian institutions represents a crucial aspect of citizenship formation. Beyond legal protection, she experienced what can be termed “institutional resilience support”; where formal structures actively enable individual resilience:

“*I felt protected, and I felt certain that when the moment comes and I need help, the help will come.”* (PA)

This comment referred to her divorce from an abusive second husband and illustrated her confidence in the agencies of the Norwegian public system, such as police and child protection services, that had supported her and enabled her to consequently rebuild her life.

These events contrast sharply with her experiences in Ukraine, where institutional support during domestic abuse situations is lacking. Her narrative concludes with a powerful testament to personal growth and empowerment:

“*So, my freedom started, school started. I had* [my own] *time when I didn't have to report to anyone what I was doing and what … decisions I made.”* (PA)

Participant A's journey exemplifies what scholars call “transformative citizenship”: where the process of becoming a citizen involves not just legal status, but a fundamental transformation of identity and agency. Her early preparation for war demonstrates what Lenette et al. (2013) describe as anticipatory resilience:

“*I was feeling a lot of pressure on me because it was I who was taking the responsibility of planning the escape because my husband said that everything would be okay.”* (PA)

Participant A's quotes reveal how forced migrants actively construct their citizenship through strategic decision-making and proactive survival strategies. This experience transformed her understanding of citizenship rights and state protection. Her subsequent engagement with education and autonomous decision-making reflects what citizenship theorists call “active citizenship”: where individuals actively participate in shaping their new social context rather than merely receiving protection.

### 3.2 Participant B: navigating intergenerational trauma and new beginnings

The second participant's story begins with childhood experiences of maternal neglect and abuse, which shaped her approach to challenging situations later in life. Her narrative provides insight into how past trauma influences refugee resilience:

“*I was afraid of her because I was thinking ‘this woman, she is my mother… she's supposed to love and care for me' … but she didn't.”* (PB)

Her wartime experience in Norway is marked by a strong desire to break cycles of trauma, particularly in her relationship with her child. Her focus on appropriate parenting in the new context shows the intersection of personal resilience and cultural adaptation.

“… [when talking to my child] *I have some reactions that I need to stop. I catch myself responding inappropriately* [to child's behavior]*... I think I understand that it's not the correct reaction … I need to try harder when I'm communicating* [with my child]*.”* (PB)

Participant B's narrative illustrates the concept of cultural citizenship—the right to be different while maintaining participation in the democratic state. PB demonstrates a complex process of intergenerational resilience negotiation, simultaneously attempting to disrupt inherited familial challenges while strategically navigating processes of social integration within the Norwegian sociocultural context:

“*I was thinking about how I wanted to live, and I didn't want to live without my husband. He would calm me down. He thought that … it was okay to cope with this situation.”* (PB)

This reflects what citizenship theorists call “participatory adaptation”: where immigrants actively negotiate between their cultural background and new social contexts. Her experience of feeling “*disconnected, because I feel like a tree that was moved but the roots are in my country”* (PB) perfectly illustrates the concept of transnational citizenship experience.

### 3.3 Participant C: transforming trauma into civic engagement

Early experiences with familial alcoholism created foundational coping mechanisms that strategically influenced Participant C's refugee adaptation. Her childhood adversity cultivated a nuanced resilience, evident in her systematic processing of war-related challenges. Initially resistant to the refugee label—“*I hate it when people call me a refugee”* (PC)—she gradually developed what Ungar (2008) identifies as adaptive identity-based resilience. Her narrative exemplifies the complex process of resistant citizenship—where individuals actively negotiate and resist imposed identities while simultaneously constructing new forms of belonging. Her reflections capture the transformative journey of adaptive integration: “*Slowly this place helped me a bit to understand that yes, the world is different.”* (PC)

The early experiences of navigating familial complexity, characterized by both love and challenge, laid the groundwork for her later resilience. The nuanced origins of her adaptive capacity are revealed in this profound statement:

“*After I was born, I had a happy upbringing with my grandparents, my mother's parents. It was a very nice environment.”* (PC)

The analysis of her narrative demonstrates how childhood experiences fundamentally shape subsequent approaches to citizenship, belonging, and personal agency.

### 3.4 Participant D: motherhood as a bridge to citizenship

A multifaceted story of resilience across various life challenges is shared by Participant D. Her account demonstrates sophisticated emotional regulation, particularly evident during the Russian occupation of her Ukrainian village:

“*I was the one who kept calm. I held myself together, and I calmed everyone down. I gave tasks to people for them to have something to do to take their minds off what was going on.”* (PD)

This capacity for emotional management while supporting others demonstrates what Sisto et al. (2019) identify as “compound resilience”: the ability to maintain functionality while simultaneously providing stability for others during crisis. Her narrative shows remarkable cognitive flexibility in adapting to multiple displacements. After fleeing from the east of Ukraine in 2014 during the earlier conflict, she rebuilt her life in another Ukrainian region, only to face displacement again in 2022. Despite experiencing severe trauma during the 36-day occupation by Russian forces, she demonstrated further resilience in her eventual adaptation to Norway:

“*Norway's such a wonderful country for me. I did not even imagine that people could live like this.”* (PD)

This participant's story also illustrates how motherhood shapes both resilience strategies and pathways to citizenship, exemplifying what can be called maternal citizenship, where civic engagement and belonging are mediated through maternal identity: “*I devoted myself to motherhood. I loved to take care of my child.”* (PD). Her decision-making consistently prioritized her children's wellbeing, from her difficult choice to temporarily leave her child to earn money in her early motherhood, to her determination to flee Ukraine with her child after experiencing bombings in the capital city.

### 3.5 Participant E: professional identity and economic citizenship

Participant E's case illustrates how professional identity can serve as a critical resilience resource during forced displacement. Her ability to balance professional obligations with displacement challenges shows sophisticated resilience strategies, particularly in maintaining personal agency during forced migration. Her experience exemplifies Brance et al. (2024) concept of resource-based resilience, where existing capabilities are leveraged for adaptation. Participant E shared her experience of managing a business installing gas measuring equipment; some of the welders left due to political instability, and she was unable to complete their specialized technical work, despite her multiple roles as lawyer, accountant, and management leader.

“*I was working night and day... that was worrying me a lot because I could not do* [her colleagues'] *job.”* (PE)

Her journey shows how professional identity can serve as a bridge to citizenship, even while processing trauma and displacement. Her gratitude for safety in Norway while maintaining concern for those left behind illustrates the transnational citizenship consciousness. The narrative demonstrates the intersection of professional identity, motherhood, and citizenship formation. Her experience demonstrates what scholars call “compound resilience”: drawing strength from multiple roles and identities.

### 3.6 Participant F: integrational citizenship formation

Participant F's narrative reveals complex interactions between past trauma and present resilience mechanisms. Her childhood normalization of confrontation created adaptive patterns that influenced her later relationships and refugee experience.

“*The shouting was normal for me. I felt like it was what was possible at that time.”* (PF)

Throughout multiple challenging relationships with abusive partners, she demonstrated a remarkable protective capacity toward her children, prioritizing their safety and wellbeing even under extreme circumstances. Her evacuation journey from Ukraine illustrates sophisticated decision-making under pressure, navigating border crossings with young children despite one child's illness and her own fears. When she describes feeling relief after crossing the border, her capacity for emotional processing even during acute stress is evident:

“*When we went through the border, it was like my whole body relaxed. All that tension went away.”* (PF)

Her adaptation to Norway illustrates the contradictory emotions inherent in displacement, where deep appreciation for safety conflicts with an overwhelming longing to return home:

“*When I went to bed it was so cozy. I didn't even unpack my bags... I wanted to go back [to Ukraine] very much.”* (PF)

Her statement “*I have nothing here and it's like starting life from the beginning”* reflects what Hutchinson and Dorsett (2012) term “protective resilience”: the ability to acknowledge profound loss while still creating security for her children in a new environment. Her narrative exemplifies “protective citizenship,” where civic engagement is driven by the imperative to safeguard children's welfare. This protective instinct extends beyond her own family to encompass her husband's children from a previous relationship, demonstrating how displacement can broaden one's sense of responsibility:

“*I understood the whole time that I could not be with him [my husband]. I was very sorry for the children, for his children.”* (PF)

The participant's concern for “his children” reveals how forced migration creates complex caregiving networks that transcend traditional family boundaries, motivating civic participation as a means of protecting vulnerable dependents.

## 4 Discussion

This study's examination of Ukrainian female refugees in Norway offers diverse insights into how citizenship is formed through daily interactions, emotional experiences, and personal agency when women navigate life after forced migration. The findings contributes to understand the ways in which identity reconstruction and resilience emerge through both the external structures provided by the host country and the internal psychological processes shaped by trauma, caregiving responsibilities, and past life experiences. The participants' autobiographical narratives built during the NET process demonstrate that their experiences of migration and settlement in Norway cannot be understood solely in terms of legal status or service access. By integrating Sobhy (2024)'s framework of lived citizenship—encompassing protection, provision, participation, and belonging the findings reveal how forced migrants negotiate their relationship with the state and construct new social identities. The six cases collectively demonstrate manifestations of resilience in forced migration contexts, supporting Pierobon (2024) conceptualization of refugee resilience as a dynamic, ongoing process rather than a fixed trait, while also aligning with Andrushko and Lanza (2024) emphasis on the reciprocal interplay between individual capacity and institutional support. The narratives illustrate how resilience operates through multiple concurrent mechanisms: transformation of pre-existing trauma into adaptive capacity, mobilization of maternal identity as a resilience resource, utilization of professional identity for integration, and navigation of intergenerational patterns of adaptation. This complex interaction between individual agency and structural support provides important insights into how the Norwegian institutional context facilitates resilience development, while simultaneously highlighting how personal agency functions as an essential component in refugees' chronological reconstruction of belonging and identity in their new environment.

The results substantiate Isin and Nielsen (2008) conceptualization of citizenship as a series of performative acts rather than a static status. This theoretical perspective illustrates the discrepancy between conventional legal-political configurations and the lived realities of refugees who actively construct citizenship through resistance and negotiation. The experiential dichotomy of refugee status—simultaneously a legal classification and a contested personal identity—reveals citizenship as a continuous negotiation involving institutional engagement and identity reconstruction. Ungar (2011) socioecological model of resilience provides a framework for understanding adaptation strategies. Findings demonstrate how resilience operates as a dynamic process of negotiation between personal capabilities and contextual opportunities, revealing compound resilience mechanisms (Ryan et al., 2008) through the simultaneous activation of multiple adaptive resources. Emotional citizenship (Askins, 2016) emerges as a fundamental element in participants' narratives, illuminating the disparity between legal status and psychological belonging. The chronological reconstruction of citizenship beyond formal status encompasses emotional attachments and psychological adaptation, with the narratives demonstrating how the modification of network relationships reshapes identity. This perspective directly challenges Marshallian conceptions of citizenship as a linear progression of rights acquisition, emphasizing instead what Fransen-Jaïbi et al. (2021) term “participatory citizenship.”

From the analysis of the narratives emerged also patterns of active engagement in rebuilding continuity with their prewar past while simultaneously situating themselves within the new socio-political scenery. This aligns with recent psychological research documenting how Ukrainian women displaced during the 2022 invasion must manage a dual burden of trauma and responsibility, often carrying the full emotional weight of supporting children and extended family under unpredictable conditions (Baran et al., 2024; Preiss et al., 2024). Such studies reinforce that women's adaptive strategies frequently emerge from the convergence of maternal identity, emotional regulation, and the need to maintain stability for others.

The research findings emphasize the significance of identity in a migration context, with a key emphasis on how individuals seek to restore a sense of continuity and coherence in their post conflict lives. Many participants expressed a desire to reconnect with their former selves through activities that grounded them, such as consuming familiar media, exchanging ideas with respected local actors, and pursuing education in their areas of specialization. This resonates emerging evidence on Ukrainian refugee mothers and professionals, which shows that reclaiming aspects of prewar identity supports emotional equilibrium and reduces the perceived loss of self often experienced in exile (Krupelnytska et al., 2025).

For all participants with children, motherhood appeared as a central pillar of identity construction, consistent with research highlighting maternal orientation among displaced Ukrainian women after 2022 (Rodríguez-Muñoz et al., 2023). Caring for children provided a stabilizing sense of purpose and structured the women's daily routines, serving simultaneously as an anchor and a burden. In addition with the fact that Ukrainian mothers under temporary protection experience intensified anxiety, chronic vigilance, and pressure to maintain emotional stability for their children (Anjum et al., 2025).

Although the women in this study shared a common experience of forced migration, their individual trajectories differ significantly. This variation underlines the concept of situational uniqueness within a shared experience of displacement. Each participant's development of resilience is shaped by personal histories, cultural expectations, family structures, and encounters with institutions. This resonates with findings among Ukrainian refugee women in Poland and the Czech Republic, where psychological resilience was found to be both situational and relational, developing in response to the specific social and institutional contexts in which women found themselves (Baran et al., 2024; Preiss et al., 2024).

Resilience emerged as a central theme, defined here as the ability to adapt successfully despite adversity. According to the participants, resilience developed through a combination of internal coping mechanisms—such as emotional reflection, maintaining hope, and seeking meaning—and external supports, including reliable social services and community support networks (Pierobon, 2024; Andrushko and Lanza, 2024). Such mechanisms of resilience appear to be vital in promoting psychological well-being and enabling individuals to entail their social identity as studied by Sobhy (2024). The findings correspond to broader research documenting that Ukrainian women displaced after 2022 engage in “layered resilience,” combining emotional self-regulation, caregiving responsibility, and proactive institutional engagement (Adedeji et al., 2025). In that perspective, the psychological literature shows that women's resilience strategies often emerge not as abstract coping mechanisms but as concrete, daily acts anchored in family protection, future planning, and maintaining dignity in the face of uncertainty (Chrzan-Detkoś and Murawska, 2023).

Another key finding is the interplay between agency and victimhood. While women acknowledged their vulnerability as refugees, they simultaneously resisted being defined by it. Many spoke of reclaiming agency through education, work, or advocacy activities. These actions can be interpreted as empowering responses that challenge passive stereotypes of refugees and emphasize their active role in shaping their futures. This pattern is well documented in recent research on Ukrainian women displaced during the ongoing war, where survivors often adopt assertive, future-oriented strategies as a way to counteract feelings of helplessness (Baran et al., 2024; Preiss et al., 2024). Agency is expressed not only in career decisions but also in everyday acts of caregiving, community-building, and negotiating bureaucratic systems—behaviors which represent small but cumulative steps toward lived citizenship (Nyers, 2015).

The participants' narratives also reveal that cultural identity plays a crucial role in shaping the meaning of citizenship (Askins, 2016). Most of the participants, as presented in the results, described the need to reconcile their sense of national belonging with the cultural expectations and norms of Norway. This negotiation reflects the fluid nature of identity in transnational contexts, where individuals maintain ties to their homeland while integrating into a new society. Studies of Ukrainian refugees consistently show that women experience strong emotional ties to Ukraine, even while actively participating in host-country institutions and communities (Krupelnytska and Morozova-Larina, 2025). This dual orientation—feeling both “here” and “there” —is not a sign of incomplete integration but a common psychological configuration among forcibly displaced women navigating uncertain futures.

Overall, the study's findings suggest that citizenship formation among Ukrainian refugee women is a multifaceted process that involves emotional, social, and structural elements (Politi et al., 2023; Erdal et al., 2018). The participants' experiences demonstrate that citizenship is constructed not only through legal recognition but through lived experiences, daily practices, and the interplay between individual agency and institutional structures (Zajdel, 2023). The integration of psychological literature on displaced Ukrainian women helps contextualize these findings by showing that identity reconstruction, emotional regulation, and maternal responsibility play fundamental roles in shaping women's engagement with citizenship processes after forced migration (Rodríguez-Muñoz et al., 2023; Chrzan-Detkoś and Murawska, 2023).

The emerged data hold implications for theoretical approaches to forced migration, calling for more meticulous models that recognize the simultaneous operation of multiple citizenship and resilience processes. The research challenges the discrepancy between policy frameworks that often treat refugees as passive recipients of aid and the lived reality of forced migrants as active agents in constructing new forms of political subjectivity (Nyers, 2015). By acknowledging the diversity of refugee experiences and the complex interplay between individual agency and structural constraints, this study contributes to formulating more effective approaches to support forced migrants in their journey toward belonging and participation.

### 4.1 Strengths and limitations of the study

A key strength of this study lies in its rich, longitudinal engagement with participants during their initial period of displacement and settlement in Norway, capturing real-time experiences of citizenship formation and resilience development rather than retrospective accounts. The multiple case study approach, incorporating six participants with diverse backgrounds and family situations, enabled both detailed individual analysis and cross-case comparison, providing nuanced insights into how Ukrainian females navigate forced migration. Additionally, the fulfillment of the study during an ongoing conflict offered unique opportunities to examine how citizenship and resilience processes unfold during active crisis situations, contributing novel theoretical understanding to forced migration scholarship.

However, limitations must be acknowledged. The Ukrainian female refugees living in Norway who participated all reside in the capital or municipalities nearby which, while providing depth of understanding, may limit generalizability to Ukrainian refugees in other parts of Norway, other host countries, and other refugee populations. The relatively recent nature of participants' displacement means that longer-term citizenship and resilience processes could not be observed. Methodological limitations include potential interpretation challenges in cross-language research and the time-bound nature of data collection during ongoing conflict. These limitations suggest opportunities for future research examining these processes across different contexts and over extended time periods.

### 4.2 Implications and recommendations for future research

Successful refugee integration requires reimagining institutional support as flexible, adaptive frameworks that recognize refugees' complex identities and capabilities. This approach demands moving beyond viewing refugees as passive aid recipients; mechanisms should be developed that validate pre-migration professional skills while providing pathways for local adaptation. Citizenship must be understood as a dynamic, ongoing process of adaptation, enabling refugees to actively construct belonging through meaningful social and professional participation. Psychological support services should adopt trauma-informed approaches that simultaneously acknowledge past experiences and promote individual agency. By implementing these principles, host societies can develop more responsive, humane approaches to refugee integration that respect individual experiences while facilitating meaningful social inclusion.

Future research should address longitudinal comparative studies tracking refugee integration processes across diverse populations and host contexts, exploring how intersectional factors such as age, professional background, and family structure influence citizenship formation. Additionally, innovative methodological approaches integrating interdisciplinary perspectives from psychology, sociology, and performance arts could provide deeper insights into the complex dynamics of resilience and belonging in forced migration contexts.

## 5 Conclusion

The study of Ukrainian female refugees in Norway reveals the complex, dynamic processes of citizenship formation and resilience in forced migration contexts. By examining individual experiences through citizenship and resilience theoretical frameworks, the research provides critical insights into how forced migrants navigate profound social transformations. The findings demonstrate that citizenship emerges as a multidimensional process of continuous negotiation, characterized by three primary mechanisms: strategic institutional engagement, identity reconstruction, and adaptive resilience. Participants actively transformed their refugee status from a passive categorization to an active process of belonging, challenging traditional understandings of citizenship as a fixed legal construct.

Theoretical contributions include an expanded conceptualization of acts of citizenship as performative processes, the identification of emotional citizenship work, and the recognition of maternal and professional identities as significant pathways to civic integration. The research reveals resilience not as a static trait, but as a dynamic, context-dependent phenomenon involving continuous adaptation and strategic navigation of new social environments. The study highlights the remarkable human capacity for adaptation, demonstrating how individuals reconstruct social identities through sophisticated strategies of resistance, negotiation, and integration. Participants' narratives illustrate how forced migrants actively engage with institutional structures, sustain transnational connections, and develop new forms of belonging while preserving core aspects of their personal and professional identities.

Taken together, this study contributes to the emerging scholarship on forced migration by demonstrating how Ukrainian women reconstruct citizenship and belonging at the intersection of trauma recovery, caregiving obligations, professional identity, and institutional interaction. The findings underscore the need for host societies to recognize the psychological dimensions of citizenship formation, ensuring that policies and practices support both structural inclusion and the emotional work of rebuilding a coherent sense of self after displacement.

## Data Availability

Anonymized and de-identified data can be made available under a reasonable request and and subject to approval of the ethical committee since the participants of the study are a vulnerable population. Requests to access the data should be directed to the corresponding author.
